# Salt-Intake-Related Behavior Varies between Sexes and Is Strongly Associated with Daily Salt Consumption in Obese Patients at High Risk for MASLD

**DOI:** 10.3390/nu15183942

**Published:** 2023-09-12

**Authors:** Bianca Heller, Florian P. Reiter, Hans Benno Leicht, Cornelia Fiessler, Ina Bergheim, Peter U. Heuschmann, Andreas Geier, Monika Rau

**Affiliations:** 1 Division of Hepatology, Department of Internal Medicine II, University Hospital Würzburg, 97080 Würzburg, Germany; 2 Institute of Clinical Epidemiology and Biometry, Julius-Maximilians-University of Würzburg, 97080 Würzburg, Germany; 3 Department of Nutritional Sciences, Molecular Nutritional Science, University of Vienna, 1040 Vienna, Austria

**Keywords:** MASLD, steatotic liver disease, salt consumption, salt-intake-related behavior

## Abstract

Background: Metabolic dysfunction-associated steatotic liver disease (MASLD) imposes a significant burden on Westernized regions. The Western diet, high in salt intake, significantly contributes to disease development. However, there are a lack of data on salt literacy and salt intake among MASLD patients in Germany. Our study aims to analyze daily salt intake and salt-intake-related behavior in MASLD patients. Methods: 234 MASLD patients were prospectively included. Daily salt intake and salt-intake-related behavior were assessed via a food frequency questionnaire (FFQ—DEGS) and a salt questionnaire (SINU). Statistical analyses were performed using SPSS. Results: Mean daily salt intake was higher in men than in women (7.3 ± 5 g/d vs. 5.3 ± 4 g/d; *p* < 0.001). There was significant agreement between increased daily salt intake (>6 g/d) and the behavioral salt index (SI) (*p* < 0.001). Men exhibited higher SI scores compared to women, indicating lower awareness of salt in everyday life. Multivariate analysis identified specific salt-intake-related behaviors impacting daily salt consumption. Conclusions: Our study reveals a strong link between daily salt intake and salt-intake-related behavior, highlighting sex-specific differences in an MASLD cohort. To enhance patient care in high-cardiovascular-risk populations, specific behavioral approaches may be considered, including salt awareness, to improve adherence to lifestyle changes, particularly in male patients.

## 1. Introduction

Western-type diets (WDs) with a high consumption of calorically rich, (ultra-) processed foods combined with chronic overnutrition and a sedentary lifestyle evoke a state of chronic metabolic inflammation [1], which contributes to diseases, such as type 2 diabetes mellitus (T2DM) as well as metabolic dysfunction-associated steatotic liver disease (MASLD). Recently, a consensus statement on a new nomenclature for fatty liver disease was published, and the term nonalcoholic fatty liver disease (NAFLD) is now replaced by MASLD [2]. MASLD is the most frequent chronic liver disease, with a global prevalence in the general population of about 25%, with a significant increase expected in the next few years [3,4].

A WD is characterized by an excessive intake of highly processed foods, including red meat, sugary drinks, snacks, cakes, and biscuits [5]. The salt content in processed foods can be more than 100-times higher in comparison to similar home-made meals, and an estimated 75% of sodium intake comes from processed or restaurant-prepared food [6]. So far, the high salt content in the WD has been mainly studied in the pathogenesis of cardiovascular diseases [7]. Here, the deleterious effect of a high-salt diet (HSD) is driven by arterial hypertension and associated with increased morbidity and mortality on a global scale [7]. The consumption of major foods and nutrients across 195 countries was recently analyzed for the Global Burden of Disease Study [8]. In this study, the worldwide sodium consumption greatly exceeded the recommended threshold, reaching 6 g of sodium per day (equivalent to 15 g of salt (NaCl) per day and 86% greater than the optimal amount) [8]. Furthermore, this analysis showed that, in 2017, more than half of diet-related deaths were attributable to a high intake of sodium [8]. Regional variations were evident, with only the Caribbean and African regions showing sodium intake within the recommended levels [8].

The consumption of ultra-processed foods (UPFs) has increased worldwide in the last few years. In the European Prospective Investigation into Cancer and Nutrition (EPIC) study, the mean energy intake of highly processed foods varied between 61% (Spain) and 79% (Germany) [9]. The short-time effect of UPF consumption was studied in a cross-over trial with 20 volunteers, who received either ultra-processed or unprocessed diets for 14 days each [10]. Diets were matched for calories, sugar, fat, fiber, and macronutrients but provided ad libitum. The UPF group consumed an additional 500 kcal/day, and BMI increased accordingly compared to the unprocessed group. There are several hypotheses regarding the possible mechanistic links between UPF consumption and the incidence of chronic diseases [11]. One of these hypotheses describes the poorer nutritional quality of UPFs, as they are characterized by a higher content of saturated fat, added-sugar energy density, and salt, along with a lower fiber and vitamin content [11]. In a cross-sectional study of 789 volunteers, a high consumption of UPFs was further associated to metabolic syndrome and, in MASLD patients, with fibrosis [12]. In a prospective Chinese study cohort, the consumption of ultra-processed foods was associated with a higher risk for MASLD after adjusting for various variables including total energy intake [13]. Another prospective study in China revealed that a high daily salt intake was associated with a higher risk of developing MASLD in the future [14]. High salt intake is a risk factor for metabolic diseases, including obesity, hypertension, dyslipidemia, and T2DM [15,16]. In preclinical MASLD models, a high-salt diet decreased antioxidant defenses and increased inflammation and fibrosis in the liver of animals [17,18]. The DASH (Dietary Approaches to Stop Hypertension) diet is characterized, amongst other things, by a sodium reduction. This diet was analyzed in a randomized clinical trial over 8 weeks in MASLD patients and showed beneficial effects on serum markers and body weight [19]. Reductions in UPF consumption and salt intake could be a strategy for the prevention of obesity and associated diseases such as MASLD.

To date, no data exist about daily salt intake or the salt-intake-related behavior of MASLD patients in Germany. The term “food literacy” describes that knowledge, skills, and behaviors are required to be put into practice for healthy diets [20]. A survey on knowledge and salt-intake-related behavior in an Italian cohort showed that the study population had a decent level of knowledge about salt but less satisfactory behavior [21]. A further gender-specific difference is observed with higher regular sodium consumption in men compared to women [22]. The current first-line treatment for MASLD patients is lifestyle change, with weight reduction and the recommendation of a Mediterranean diet [23]. But, only a few patients show long-term adherence to these recommendations [24,25].

The main focus of this study was to prospectively evaluate salt-intake-related behavior in a well-characterized cohort of MASLD patients and analyze its association with their daily salt intake. Gender-specific differences in salt-related behavior were further analyzed, as regular salt consumption is reported to be higher in men. Specific salt related behavior could be further addressed in a personalized manner of nutrition recommendations for MASLD patients to facilitate long-term adherence.

## 2. Materials and Methods

### 2.1. Cohort Characteristics

In this prospective single-center study, 234 patients were included after providing informed consent from 04/2021–01/2022. The study protocol was implemented in accordance with Good Clinical Practice Guidelines and the Declaration of Helsinki. The study was reviewed and approved by local Ethics Committee (AZ-188/17-mk). All patients were >18 years old. MASLD was diagnosed either via controlled attenuation parameter (CAP) measurement or liver histology. Every MASLD patient included in the study had at least one cardiometabolic risk factor, as per the defined criteria [2]. Patients with significant alcohol consumption (women > 20 g/day and men > 30 g/day) and other concomitant chronic liver diseases were excluded. Thus, 107 patients had histologically diagnosed MASLD, and 15 patients underwent liver biopsy without histological diagnosis of MASLD but with a clinical diagnosis of MASLD. Further, 90 patients were included before undergoing bariatric surgery.

### 2.2. Food Frequency Questionnaire

To analyze the usual food consumption of the patient cohort, a self-administered and semi-quantitative food frequency questionnaire (FFQ) based on the German Health Examination Survey for Adults 2008–2011 (DEGS) with slight modifications was used [26]. Specifically, in addition to the frequency and portion size of 53 ordinary German food products, long-term eating behavior was assessed by extending the assessment time. Furthermore, three food products, including pretzels, rolls, or pretzels, canned cold fish, and smoked salmon, were added for more accurate salt detection. In addition, the FFQ was supplemented with the question of how often the patient prepares a meal from convenience foods. Seven questions of the DEGS-FFQ concerning low-fat products, deep-fried meals, or beverage consumption were removed as they did not fit the purpose of this study.

### 2.3. Daily Salt Intake

To calculate daily salt intake, the daily consumption of each food in grams per day was determined using a Healthy Eating Index developed specifically for the DEGS study (HEI-DEGS1) [27]. Average consumption of one food group was calculated on the basis of consumption frequency in relation to opportunities per 28 days and portion size of each product. The procedure for missing responses was adapted to that of the HEI-DEGS1: If both details for a question (frequency of consumption and portion size) were missing, this food was considered as “never consumed”. If the portion size information (frequency of consumption available) was the only missing data, it was substituted with the average information of the corresponding food for both sexes. The salt content of the individual food products and dishes was taken from a food table. For this study, “Die Nährwerttabelle” was used [28]. It contains the most frequently consumed foods and dishes in Germany, based on consumption studies. Each salt content per 100 g of ready-to-eat food is listed in milligrams. To calculate the salt content of each consumed food product in grams per day, the following equation was used:

daily salt intake by one food product [g/d] = (daily consumption of the food product [g/d] × it’s amount NaCl per 100 g × 10^−3^ [g])/100 [g]

Beverages were excluded for this analysis as they do not account for a relevant contribution to salt consumption. To obtain the daily salt intake of each patient, the calculated salt amounts of the individual food products were added up.

### 2.4. Salt-Intake-Related behavior

Salt-intake-related behavior of MASLD patients was assessed by adapting questions from the Italian Salt Literacy Questionnaire of the SINU (Società Italiana di Nutrizione Umana) [21]. The questions are self-administered multiple-choice questions about behavior and knowledge related to salt intake. In this study, 14 SINU questions were used, from which a salt index (SI) was calculated. The number of response options was standardized to 3-point Likert scale for this study. Points were assigned from 0 to 2. All questions used and their scores are listed in Table A1. For the calculation of the SI, all scores of the individual salt behavior questions were added up. Finally, a score between 0 and 28 points could be achieved. The lower the SI, the higher the awareness of salt in the patient’s everyday life. In addition to the SINU questions, the “Würzburg Salt Questionnaire” comprised 5 extra questions that evaluated the frequency of meal preparation using basic ingredients/fresh foods or convenience foods for each participant. Additionally, the questionnaire inquired about the presence of a saltshaker during meals and the frequency of restaurant or cafeteria visits before and during the COVID-19 pandemic. The corresponding response options and scores are shown in Table A2.

### 2.5. Statistical Analysis

To analyze the association between salt-related behavior and the daily salt consumption, the statistical software IBM^®^ SPSS^®^ Statistics, Version 28.0.0.0 (190) for Windows (IBM Corp.) was used. The Kruskal–Wallis test was performed to analyze differences between two or more groups of the salt behavior questions. Subsequently, pairwise comparisons were calculated using the Mann–Whitney-U-test. Finally, to find out which salt behavior questions have the greatest influence on daily salt intake, a multiple linear regression with backward selection was performed as the requirements for this multivariate analysis were met. Pearson’s chi-square test and Fisher–Freeman–Halton exact test were used for the gender-specific analysis of the data. The listed results include medians with interquartile ranges (IQRs), mean values with standard deviations, frequencies, and *p*-values of asymptotic significances. A significance level of <0.05 was set. Deviating *n*-numbers in the results are due to missing items.

## 3. Results

### 3.1. Patient Characteristics

In this prospective study, 234 MASLD patients were included, 56.4% women and 43.6% men. The median age was 55 ± 17 years, and BMI was 32.8 ± 9.9 kg/m^2^. MASLD patients had a high prevalence of arterial hypertension (58.6%), T2DM (37.7%), and dyslipidemia (23.0%); 107 patients had histologically confirmed MASLD (82 with MASH and 25 with MAFL); and 127 patients received a clinical diagnosis via CAP measurement. Median serum ALT was slightly elevated, with 36 ± 35 U/L. Further laboratory findings are depicted in Table 1, including the median CAP value of 320 ± 90 dB/m and stiffness of 6.0 ± 3.7 kPa.

### 3.2. Lower Salt Intake and Higher Salt Awareness

**Figure 1:**
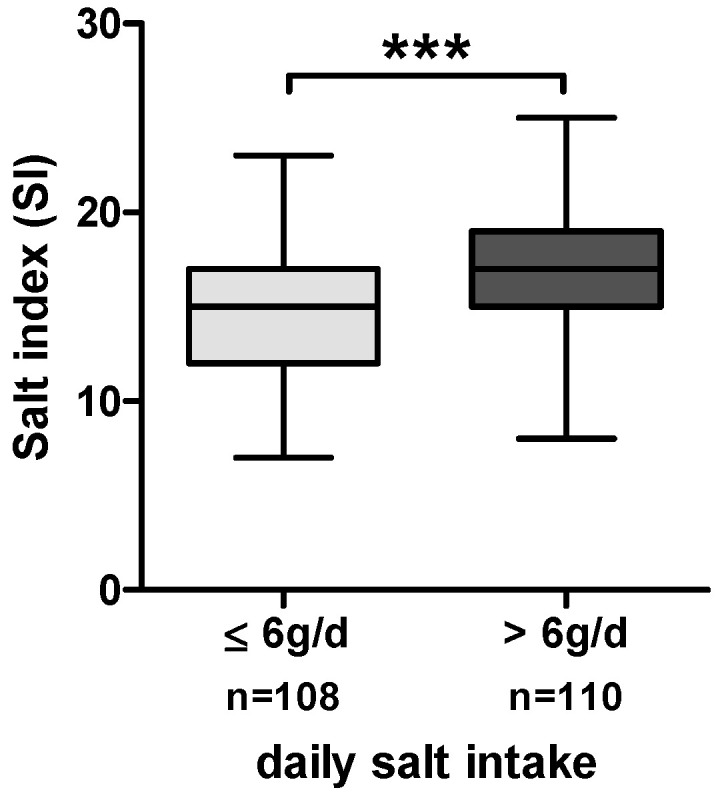
Boxplots of SI in the total cohort of MASLD patients stratified by daily salt intake (≤6 g/d and >6 g/d). *** = *p* ≤ 0.001.

The calculated daily salt intake in this cohort of MASLD patients was 6.0 ± 3.8 g/d (median ± IQR). The daily salt intake was subsequently analyzed together with specific salt-related behavior. The mean salt index (SI) reflecting the salt-related behavior as the sum of the 14 single questions was 16 ± 3.5 points (median ± IQR) (range from 0 = high awareness to 28 = low awareness). To examine SI in relation to daily salt intake, the cohort was divided into two groups. The threshold between the two groups was selected based on the median daily salt intake in our cohort of 6 g/d. As shown in Figure 1, patients with lower daily salt intake also had significantly lower SI values (*p* < 0.001, *t*-test) and, thus, showed higher awareness of salt in daily life. Daily salt intake in the group of patients with ≤6 g/d ranged from 1.1 g/d to 6 g/d (mean ± SD: 4.2 ± 1.2 g/d), while in the group of patients with >6 g/d, it ranged from 6 g/d to 28 g/d (mean ± SD: 9.7 ± 4.3 g/d).

**Figure A1:**
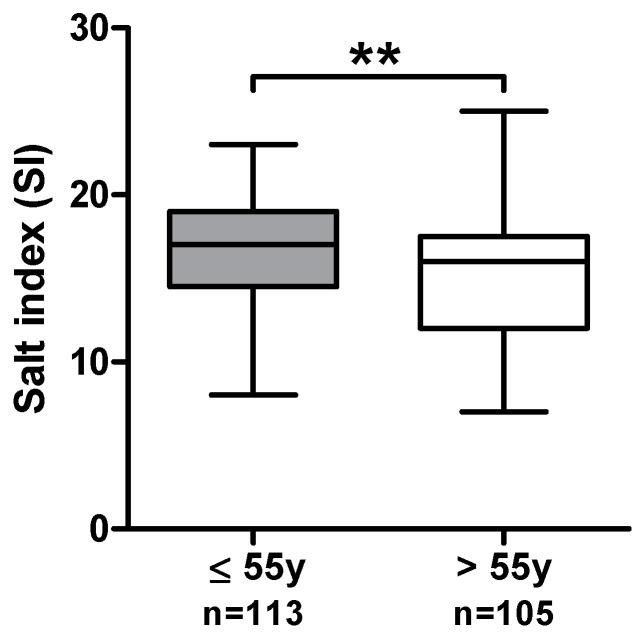
Boxplots of SI in the total cohort of MASLD patients stratified by age (≤55 years and >55 years). ** = *p* < 0.01.

Given that daily salt intake can vary across one’s lifetime, we conducted an analysis of daily salt intake in relation to age. No correlation was detected between daily salt intake and age (Spearman’s Rho = −0.037, *p* = 0.577). However, a minor negative correlation between age and SI, signifying increasing salt awareness with age, was evident (Spearman’s Rho = −0.189, *p* = 0.005). To investigate the age-dependent effects further, we divided the cohort into two groups based on the median age of 55 years within our cohort. Patients aged > 55 years exhibited a lower median SI of 16 compared to their younger counterparts (≤55 years, median SI = 17) (Appendix A Figure A1).

### 3.3. Specific Salt-Intake-Related Behavioral Patterns and Low Salt Intake

**Figure 2:**
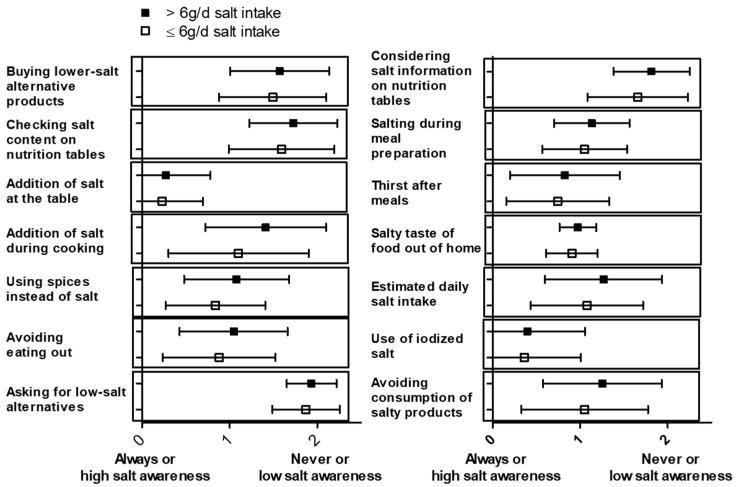
Mean ± SD are shown for each salt-intake-related behavior question for the group with high (>6 g/d, *n* = 114) and low (≤6 g/d, *n* = 120) daily salt intake.

**Figure 3:**
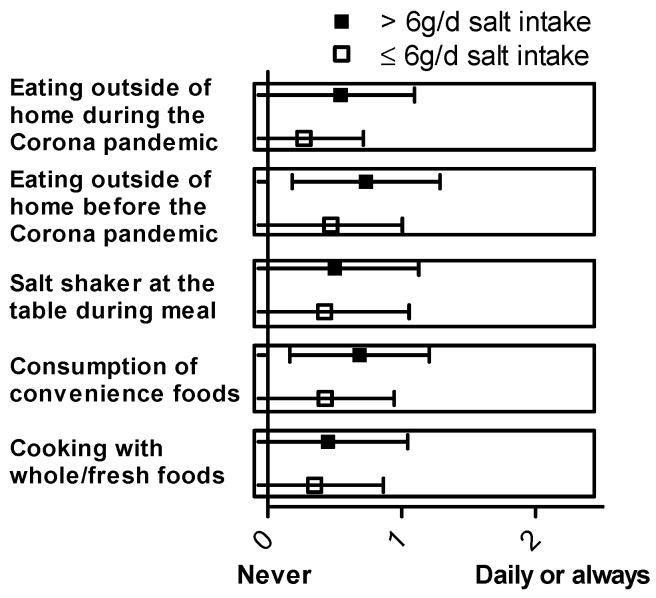
Mean ± SD are depicted for each item of the “Würzburg salt questionnaire” for the group with high (>6 g/d, *n* = 114) and low (≤6 g/d, *n* = 120) daily salt intake.

**Figure A2:**
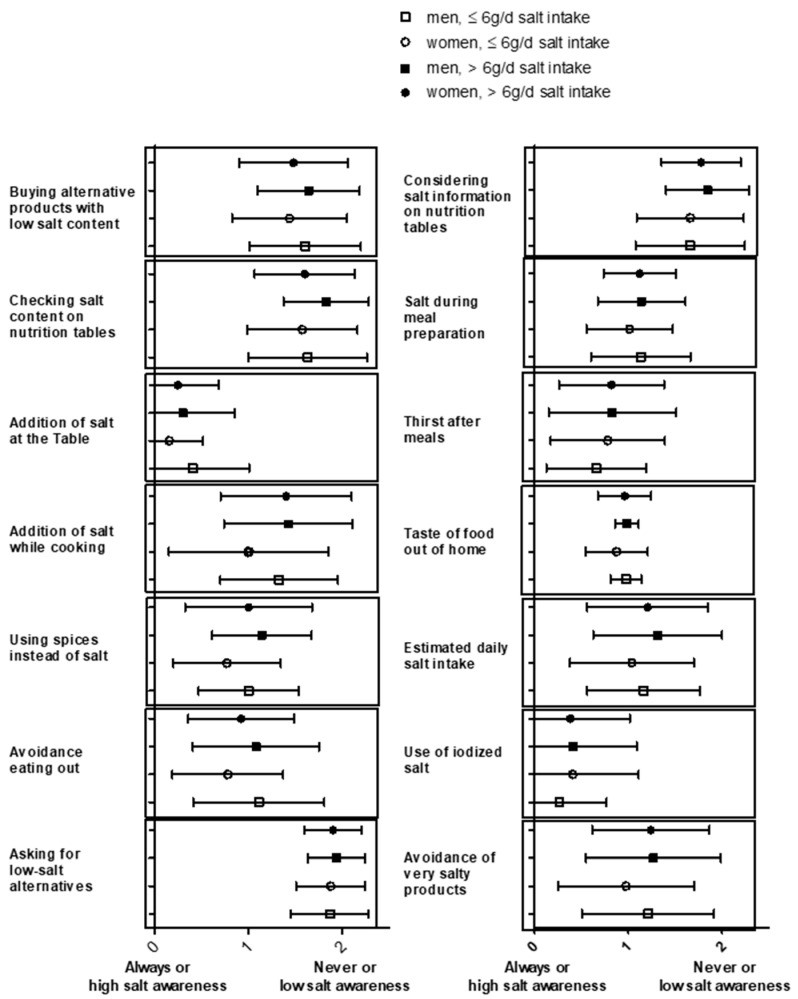
Gender-specific analysis of salt-related behavior questions. Mean ± SD are shown for each salt-intake-related behavior question for the group with high (>6 g/d, *n* = 114) and low (≤6 g/d, *n* = 120) daily salt intake and further separated by gender.

Further analysis of the single questions for the salt index (SI) addressing specific salt-intake-related behavior was performed. Figure 2 presents the mean ± SD of responses on the Likert scale for each question related to salt-intake-related behavior. A gender-specific analysis of these data is shown in Figure A2. To perform a statistical analysis, a Kruskal–Wallis test was used, comparing daily salt intake with the responses to the questions about salt-intake-related behavior. The following behavioral patterns were significantly associated with daily salt intake: considering salt information on nutrition tables (*p* = 0.004), no addition of salt during cooking (*p* = 0.009), using spices instead of salt (*p* = 0.022), checking salt content on nutrition tables (*p* = 0.023), avoiding eating out (*p* = 0.018), avoiding consumption of salty products (*p* = 0.028), and salty taste of food out of home (*p* = 0.030). In contrast, no significant association with daily salt intake was observed for the following behavioral questions: estimated daily salt intake (*p* = 0.183), salting during meal preparation (*p* = 0.415), addition of salt at the table (*p* = 0.457), buying lower-salt alternative products (*p* = 0.233), thirsting after meals (*p* = 0.711), using iodized salt (*p* = 0.754), and asking for low-salt alternatives (*p* = 0.253). The analysis of the “Würzburg Salt Questionnaire” is depicted in Figure 3, showing the mean ± SD of responses on the Likert scale for each question. To conduct the statistical analysis, a Kruskal–Wallis test was once again used to compare daily salt intake with the responses to the “Würzburg Salt Questionnaire”. The statistical analysis revealed a significant association between daily salt intake and the consumption of convenience foods (*p* = 0.001). This association was also seen with eating away from home, e.g., in restaurants or cafeterias, before (*p* = 0.001) and during the COVID-19 pandemic (*p* < 0.001). There was no significant association with patients’ salt intake when asked about the frequency of cooking with whole/fresh foods (*p* = 0.638) and when asked about the saltshaker at the table during a meal (*p* = 0.437). The summary of the results of the “Würzburg Salt Questionnaire” can be found in Table A2.

### 3.4. Multivariate Analysis—The Influence of Salt-Specific Salt-intake-related behavior on Salt Intake

After the analysis of single salt questions in relation to daily salt intake, a multivariate model was created to determine which salt behavior questions have the greatest influence on salt intake. The salt-intake-related behavior questions and the “Würzburg salt questionnaire” were analyzed together. For our research question, a multiple linear regression model including 208 patients’ data with backward selection was used, which was statistically significant ((F(6, 201) = 7.59, *p* < 0.001), with an R^2^ of 0.185 (corrected R^2^ = 0.160)). All 19 salt questions were included in the analysis, and after selection (pout(.10)), as appropriate, the final model contained six salt questions: consumption of convenience foods, considering salt information on nutrition tables, salty taste of food out of home, use of iodized salt, salting during meal preparation, and eating outside of home (restaurants or cafeterias) during the COVID-19 pandemic. Four questions represented significant factors influencing the prediction of daily salt consumption in grams per day: consumption of convenience foods (b = 0.867; CI 0.183–1.551; *p* = 0.013), considering salt information on nutrition tables (b = 0.977; CI 0.247–1.707; *p* = 0.009), salting during meal preparation (b = 0.504; CI 0.007–1.001; *p* = 0.047), and eating outside of home (restaurants or canteens) during the COVID-19 pandemic (b = 1.017; CI 0.298–1.735; *p* = 0.006). The questions about the salty taste of food out of home (*p* = 0.084) and use of iodized salt (*p* = 0.097) did not contribute significantly to the model (Table 2).

### 3.5. Gender-Specific Differences in Salt Consumption and Salt-Intake-Related Behavior

Our study focused on gender-specific differences in salt consumption and salt-intake-related behavior. In this cohort, slightly more women than men were included (56.4% women and 43.6% men). Women had significantly higher BMI compared to men (35.0 ± 12.1 kg/m^2^ vs. 30.5 ± 8.2 kg/m^2^, *p* = 0.001). Non-invasive liver stiffness measurements via Fibroscan and liver fat quantification via CAP were higher in men (men: 6.8 ± 4.0 kPa vs. women: 5.7 ± 4.2 kPa, *p* = 0.019; men: 329 ± 93 dB/m vs. women: 313 ± 100 dB/m vs., *p* = 0.039).

**Figure 4:**
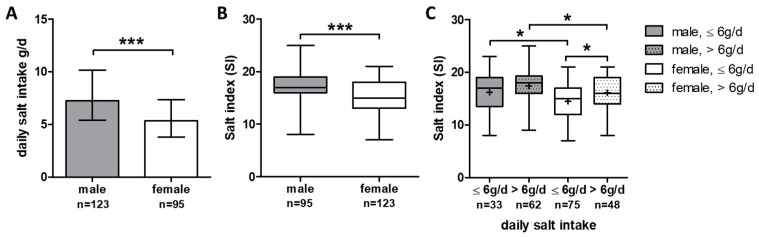
Gender-specific differences in daily salt intake and salt-intake-related behavior: (**A**) gender-specific daily salt intake shown as bars with median ± IQR. (**B**) Boxplots of salt index in men and women; (**C**) boxplots of salt index in men and women with low and high daily salt intake (≤6 g/d, >6 g/d). *** *p* ≤ 0.001, * *p* < 0.05.

Furthermore, men had higher daily salt intake 7.3 ± 4.8 g/d compared to women (5.3 ± 3.5 g/d, (*p* < 0.001); Figure 4A). According to the daily salt intake, men showed higher SI scores compared to women, reflecting a lower awareness of salt-intake-related behavior in everyday life (*p* < 0.001) (Figure 4B). A comprehensive analysis of gender-specific salt-intake-related behavior in conjunction with daily salt intake revealed a significant difference, indicating higher SI (meaning lower salt awareness) in men in both the high (>6 g/d) and low (≤6 g/d) daily salt intake groups compared to women (Figure 4C). Only in women was the difference regarding SI between high and low daily salt intake groups statistically significant (Figure 4C).

In our cohort, women showed different salt-intake-related behavior than men. Specifically, women reported that they read the salt content on nutrition tables more often (*p* = 0.002), used spices instead of salt (*p* = 0.004), and avoided eating out (*p* = 0.002), whereas men more frequently used salt in cooking (*p* = 0.005) or salt at the table (*p* = 0.016). Regarding the Würzburg salt questions, women more often reported that they cooked with whole/fresh foods (*p* = 0.005), whereas men showed a higher frequency in the preparation of convenience foods (*p* < 0.001) and the frequency of restaurant visits before (*p* < 0.001) and during (*p* < 0.001) the COVID-19 pandemic. No gender difference could be found when asked about the saltshaker at the table during a meal.

## 4. Discussion

The Western diet is characterized by its high salt content and the prevalence of highly processed foods. The consumption of such highly processed foods and adherence to a Western-type diet are significant risk factors contributing to the pathogenesis of MASLD. Growing evidence indicates that patients with MASLD are at substantial risk for the development of hypertension, coronary heart disease, and cardiac arrhythmias, which clinically result in increased cardiovascular morbidity and mortality [29]. High salt intake is associated with hypertension and risk for cardiovascular diseases, but its role in the prevention or treatment of MASLD is not known. As lifestyle changes are the primary therapeutic goal in MASLD patients and adherence to these goals is difficult to achieve, nutritional behavior has a major impact on the treatment of these patients.

In this study, daily salt intake together with salt-intake-related behavior were analyzed in a cohort of obese MASLD patients. Increased daily salt intake was linked to reduced awareness of salt consumption in everyday life. In a multivariate linear regression model, eating away from home, considering salt information on nutrition tables, consumption of convenience foods, and salting during meal preparation had the highest impact on daily salt intake. Sex-specific differences were observed, with higher daily salt intake in men compared to women, together with lower salt-intake-related behavior in men.

Daily salt intake with 6.0 g/d in our cohort (men 7.3 ± 4.8 g/d compared to women 5.3 ± 3.5 g/d) was lower than estimated in the general German Health Interview and Examination Survey for Adults (DEGS; first wave DEGS1 2008–2011), with 10.4 g/d in men and 9.2 g/d in women in the age group of 50–59 years [30]. In the DEGS study, daily salt intake exceeded 10 g/day for almost 50% of men and 40% of women [31]. However, it is important to note that the assessment of daily salt intake differed between the DEGS study and our cohort. In the DEGS study, salt intake was estimated using urinary sodium excretion (spot urine), and it was assumed that renal sodium excretion was equivalent to salt intake. In contrast, our study calculated sodium intake based on a comprehensive FFQ, which may have led to observed differences due to the potential under-reporting of salt intake within our patient cohort. These differences are in line with the significant lower estimated daily salt intake of 8.7 g/d for men and 6.3 g/d for women in a German national consumption study (Nationale Verzehrstudie II) based on diet history interviews or 24 h dietary recalls [32]. The daily salt intake observed within our MASLD patient cohort surpassed the WHO-recommended cut off (<5 g/day) [33]. The German Nutrition Society (Deutsche Gesellschaft für Ernährung e.V., DGE) offers an approximate value for salt intake in Germany of up to 6 g of table salt per day [30]. The median salt consumption identified in our study was 6 g/d, which is the upper limit of DGE’s suggested salt intake, but men are still above this limit compared to women.

Specific salt-intake-related behaviors were found to correlate with the detected daily salt intake in our cohort. The individual salt index (SI) was calculated based on the behavioral questions. Men exhibited higher daily salt intake and SI scores compared to women, indicating a greater consumption of salt and lower awareness of salt in men. In the original study using the SINU salt questionnaire, women outperformed men in both knowledge and behavior categories related to salt, highlighting the gender-specific aspect of nutritional behavior [21]. When examining specific salt-intake-related behavioral questions, we observed a significant association with lower daily salt intake for the following aspects: considering salt information on nutrition tables, salting during meal preparation, using spices instead of salt, checking salt content on nutrition tables, avoiding eating out, avoiding consumption of salty foods, and salty taste of food out of home. In the Italian analysis of behavioral questions of the SINU salt questionnaire, the majority of participants reported reducing or avoiding the consumption of salty foods. Additionally, they mentioned refraining from adding salt while cooking or eating [21]. These statements align with the specific salt-intake-related behaviors observed in our cohort. Salt awareness was also assessed in another study using a 68-item, self-administered questionnaire in a population of 141 individuals in Switzerland, specifically among individuals in a workplace setting [34]. In a multiple linear regression model exploring the relationship between health literacy, food literacy, and the variable ‘salt content impacting food/menu choice’ with salt intake, only the variable ‘salt content impacting food/menu choice’ showed a significant association with salt intake, while health literacy and food literacy did not demonstrate significant associations. Participants indicating that salt content influences their decision to purchase/choose a food or menu item had a 1.5 g lower daily salt intake [34]. In our cohort, 23 percent of patients reported that they paid attention to the salt content on nutrition tables, and this statement was also found to be significant in the multivariate analysis of this study. In a Spanish cross-sectional analysis among children and their parents, “Checking sodium content on food labels and the use of table salt by the children or father” was associated with a lower preference for salty foods. In this study, the majority of families (59%) stated that they never look at the sodium content on food labels [35]. Awareness of salt content on food and nutrition labels appears to be a crucial indicator of high salt-intake-related behavior, but it is only practiced by a minority of the analyzed populations.

In our cohort, we observed sex-specific differences not only in daily salt intake but also in salt-intake-related behavior, with men showing higher daily salt intake and lower salt awareness compared to women. Women are reported to have a 9–11% lower salt intake worldwide, which is suggested to be associated with their lower calorie intake [36]. In NHANES data, an age–gender interaction for adding table salt suggests that younger women (<30 years) tend to add salt more frequently than men, but after age 30, men more often add salt compared to women [36]. In our cohort, we also observed an age-dependent effect on salt-related behavior, indicating higher salt awareness with increasing age. However, this effect was not reflected in daily salt intake. In addition to the awareness of salt-intake-related behavior, an Australian survey identified another factor contributing to the emergence of the gender difference. This survey, which included 530 participants, revealed that female participants had lower levels of salt taste beliefs, and these beliefs had the strongest positive association with salt use [37]. Sex-specific differences in salt-intake-related behavior are observed worldwide and seem to be associated with age and salt tase beliefs.

There are certain limitations in our study that should be acknowledged. The daily salt intake was calculated using a detailed FFQ, but no measurements of sodium in 24 h urine collections were taken. As a result, intake-related bias must be considered when interpreting and comparing our data. Our analysis is based on a cohort of MASLD patients without a comparative analysis of a matched cohort of healthy obese people without steatotic liver disease. Consequently, the presented findings concentrate on variations in salt-related behavior and gender-specific distinctions within the MASLD patient cohort, without drawing comparisons to healthy controls. Additionally, our patient cohort originates from a tertiary center with specific patient selection. Larger multicentric cohort studies are suggested to validate these findings.

WHO Member States have agreed to reduce the global population’s intake of salt by a relative 30% by 2025, as reducing salt intake has been identified as one of the most cost-effective measures countries can take to improve population health outcomes [33]. The WHO’s country support package for the European region aims to accelerate salt reduction efforts and encompasses specific objectives, including raising population awareness about salt intake, empowering the public with actionable knowledge, and providing targeted assistance for reducing salt consumption [38]. To attain these objectives, it is essential to explore personalized and gender-specific nutritional approaches that can effectively drive behavioral changes in patients.

To conclude, our study demonstrates a robust association between daily salt intake and salt-intake-related behavior, highlighting sex-specific differences within an MASLD cohort of obese patients. Sex-specific behavioral nutrition patterns should be validated and analyzed in future studies to be considered for individualized nutritional treatment, which remains the first-line approach for managing MASLD patients. Given that cardiovascular diseases are the leading cause of mortality within this patient population, it is crucial to utilize all available strategies to promote adherence to lifestyle modifications. Therefore, it is essential to explore various avenues for improving patient care, including the incorporation of tailored behavioral approaches to enhance adherence to these lifestyle changes.

## Figures and Tables

**Table 1:** Patient characteristics.

Cohort	
Sex m/f	102/132
Age in years (median ± IQR)	55 ± 17
BMI in kg/m^2^ (median ± IQR)	32.8 ± 9.9
Disesases	
Arterial Hypertension % (*n*)	58.6 (116)
Diabetes Mellitus Type II % (*n*)	37.7 (75)
Hyperlipidemia % (*n*)	23.0 (46)
Hypertriglyceridemia % (*n*)	5.5 (11)
Coronary Heart Disease % (*n*)	3.5 (7)
Clinical findings in MASLD patients	
Diagnosis histological/clinical	107/127
ALT in U/L (median ± IQR)	35.5 ± 35
AST in U/L (median ± IQR)	30.0 ± 18
GGT in U/L (median ± IQR)	44.1 ± 66
CAP in dB/m (median ± IQR)	320 ± 90
Stiffness in kPa (median ± IQR)	6.0 ± 3.7

**Table 2:** Results of the multiple linear regression, and the influence of salt questions on salt consumption.

	Dependent Variable: Daily Salt Intake [g/d]							
Coefficients	b	SE	β	T	95% CI		*p*	S
					LL	UL		
(Constant)	1.837	0.972		1.890	−0.079	3.753	0.060	
Consumption of convenience foods	0.867	0.347	0.166	2.499	0.183	1.551	0.013	*
Considering salt information on nutrition tables	0.977	0.370	0.173	2.639	0.247	1.707	0.009	**
Salty taste of food out of home	1.228	0.707	0.115	1.736	−0.167	2.622	0.084	
Use of iodized salt	0.485	0.291	0.112	1.669	−0.088	1.059	0.097	
Salting during meal preparation	0.504	0.252	0.135	1.999	0.007	1.001	0.047	*
Eating outside of home (restaurants or canteens) during the COVID-19 pandemic	1.017	0.364	0.190	2.791	0.298	1.735	0.006	**

b = regression coefficient, SE = standard error, β = standardized coefficient, T = T-value, 95% KI = Confidence interval with lower and upper limit, *p* = significance value, S = significance for * *p* ≤ 0.05, ** *p* ≤ 0.01.

## Data Availability

The data presented in this study are available on request from the corresponding author. The data are not publicly available due to confidentiality reasons.

## Appendix A

**Table A1:** Salt-related behavior questions, their scores, and the frequencies of the answer options. Deviating *n*-numbers in the results are due to missing items.

Salt-Intake-Related Behavior Questions	Score	*N* (%)	*N* (%)
Daily salt intake		≤6 g/d	>6 g/d
Does the information about salt quantity reported on nutrition tables affect your food choices when grocery shopping?		*N* = 120	*N* = 113
- Never	2	85 (70.8)	94 (83.2)
- Sometimes	1	29 (24.2)	17 (15.0)
- Always	0	6 (5.0)	2 (1.8)
When you cook/eat a meal, you add salt:		*N* = 119	*N* = 114
- both while cooking and at the table	2	17 (14.3)	19 (16.7)
- only during preparation/only at the table	1	91 (76.5)	91 (79.8)
- neither during preparation nor at the table	0	11 (9.2)	4 (3.5)
Are you thirsty after meals?		*N* = 120	*N* = 114
- Always	2	9 (7.5)	14 (12.3)
- Sometimes	1	71 (59.2)	66 (57.9)
- Never	0	40 (33.3)	34 (29.8)
When eating away from home to me food tastes:		*N* = 117	*N* = 112
- Flavorless	2	0 (0)	1 (0.9)
- Normal	1	106 (90.6)	107 (95.5)
- Salty	0	11 (9.4)	4 (3.6)
In conclusion, in your opinion, how much is your daily salt intake? Taking into account the foods you buy and the ones you personally cook (including the salt added during preparation and/or after cooking)?		*N* = 118	*N* = 113
- more than 20 g	2	29 (24.6)	44 (38.9)
- between 10 and 20 g			
- between 5 and 10 g	1	69 (58.5)	55 (48.7)
- less than 5 g	0	20 (16.9)	14 (12.4)
Do you usually use iodized salt for seasoning or cooking?		*N* = 119	*N* = 114
- Never	2	11 (9.2)	11 (9.6)
- Sometimes	1	21 (17.7)	23 (20.2)
- Always	0	87 (73.1)	80 (70.2)
I avoid eating, or I reduce the consumption of products rich in salt		*N* = 119	*N* = 114
- Never	2	34 (28.6)	44 (38.6)
- Sometimes	1	57 (47.9)	55 (48.2)
- Always	0	28 (23.5)	15 (13.2)
I buy alternative products, with a low salt content		*N* = 118	*N* = 114
- Never	2	65 (55.1)	69 (60.5)
- Sometimes	1	46 (39.0)	41 (36.0)
- Always	0	7 (5.9)	4 (3.5)
I read the salt content on the nutrition tables		*N* = 120	*N* = 114
- Never	2	78 (65.0)	86 (75.4)
- Sometimes	1	35 (29.2)	25 (21.9)
- Always	0	7 (5.8)	3 (2.6)
Salt at the table		*N* = 116	*N* = 113
- I always add salt at the table.	2	2 (1.7)	3 (2.7)
- I add salt at the table sometimes.	1	23 (19.8)	25 (22.1)
- I add no, or very little salt at the table.	0	91 (78.4)	85 (75.2)
Salt while cooking		*N* = 118	*N* = 114
- I always add salt while cooking.	2	44 (37.3)	60 (52.6)
- I add salt while cooking sometimes.	1	42 (35.6)	41 (36.0)
- I don’t add salt while cooking (or only very little)	0	32 (27.1)	13 (11.4)
I use spices instead of salt in cuisine		*N* = 118	*N* = 113
- Never	2	11 (9.3)	25 (22.1)
- Sometimes	1	77 (65.6)	72 (63.7)
- Always	0	30 (25.4)	16 (14.2)
I avoid going out to eat out too often		*N* = 118	*N* = 114
- Never	2	18 (15.3)	23 (20.2)
- Sometimes	1	68 (57.6)	69 (60.5)
- Always	0	32 (27.1)	22 (19.3)
If I eat out, I ask for low-salt options		*N* = 117	*N* = 113
- Never	2	104 (88.9)	105 (92.9)
- Sometimes	1	11 (9.4)	7 (6.2)
- Always	0	2 (1.7)	1 (0.9)

**Table A2:** “Würzburg Salt Questionnaire”, their scores, and the frequencies of the answer options. Deviating *n*-numbers in the results are due to missing items.

**Würzburg Salt Questionnaire**	**Score**	***N* (%)**	***N* (%)**
Daily salt intake		≤6 g/d	>6 g/d
How often a week do you prepare a hot meal (lunch or dinner) yourself from basic ingredients/fresh foods?		*N* = 120	*N* = 114
- Daily	0	80 (66.7)	69 (60.5)
- 5–6 times per week			
- 3–4 times per week	1	38 (31.6)	39 (34.2)
- 1–2 times per week			
- Never	2	2 (1.7)	6 (5.3)
How often a week do you prepare a hot meal (lunch or dinner) yourself from convenience foods?		*N* = 119	*N* = 114
- Daily	2	1 (0.8)	3 (2.6)
- 5–6 times per week			
- 3–4 times per week	1	49 (41.2)	72 (63.2)
- 1–2 times per week			
- Never	0	69 (58.0)	39 (34.2)
Do you have a saltshaker at your table when you eat a meal?		N = 120	N = 114
- Always	2	9 (7.5)	8 (7.0)
- Sometimes	1	33 (27.5)	41 (36.0)
- Never	0	78 (65.0)	65 (57.0)
How many times a week did you eat away from home (e.g., restaurant, canteen) before the COVID-19 pandemic?		N = 119	N = 113
- Daily	2	2 (1.7)	6 (5.3)
- 5–6 times per week			
- 3–4 times per week	1	52 (43.7)	71 (62.8)
- 1–2 times per week			
- Never	0	65 (54.6)	36 (31.9)
How many times a week now, during the COVID-19 pandemic, do you eat away from home (e.g., delivery/pickup, restaurant/canteen)?		N = 119	N = 113
- Daily	2	0 (0)	2 (1.8)
- 5–6 times per week			
- 3–4 times per week	1	32 (26.9)	56 (49.5)
- 1–2 times per week			
- Never	0	87 (73.1)	55 (48.7)
